# Effects of Pharmacogenomic Testing in Clinical Pain Management: Retrospective Study

**DOI:** 10.2196/32902

**Published:** 2022-05-03

**Authors:** Christian Tagwerker, Mary Jane Carias-Marines, David J Smith

**Affiliations:** 1 Alcala Testing and Analysis Services San Diego, CA United States

**Keywords:** pharmacogenomics, pain management, drug-drug interaction, DDI, pharmacy, prescriptions, genetics, genomics, drug-gene interaction, pain

## Abstract

**Background:**

The availability of pharmacogenomic (PGx) methods to determine the right drug and dosage for individualized patient treatment has increased over the past decade. Adoption of the resulting PGx reports in a clinical setting and monitoring of clinical outcomes is a challenging and long-term commitment.

**Objective:**

This study summarizes an extended PGx deep sequencing panel intended for medication dosing and prescription guidance newly adopted in a pain management clinic. The primary outcome of this retrospective study reports the number of cases and types of drugs covered, for which PGx data appears to have assisted in optimal drug prescription and dosing.

**Methods:**

A PGx panel is described, encompassing 23 genes and 141 single-nucleotide polymorphisms or indels, combined with PGx dosing guidance and drug-gene interaction (DGI) and drug-drug interaction (DDI) reporting to prevent adverse drug reactions (ADRs). During a 2-year period, patients (N=171) were monitored in a pain management clinic. Urine toxicology, PGx reports, and progress notes were studied retrospectively for changes in prescription regimens before and after the PGx report was made available to the provider. An additional algorithm provided DGIs and DDIs to prevent ADRs.

**Results:**

Among patient PGx reports with medication lists provided (n=146), 57.5% (n=84) showed one or more moderate and 5.5% (n=8) at least one serious PGx interaction. A total of 96 (65.8%) patients showed at least one moderate and 15.1% (n=22) one or more serious DGIs or DDIs. A significant number of active changes in prescriptions based on the 102 PGx/DGI/DDI report results provided was observed for 85 (83.3%) patients for which a specific drug was either discontinued or switched within the defined drug classes of the report, or a new drug was added.

**Conclusions:**

Preventative action was observed for all serious interactions, and only moderate interactions were tolerated for the lack of other alternatives. This study demonstrates the application of an extended PGx panel combined with a customized informational report to prevent ADRs and improve patient care.

## Introduction

Over the last decades, there has been considerable growth in the use of pharmacogenomic (PGx) testing due to increased awareness of patients developing moderate to serious adverse drug reactions (ADRs) attributed to individual genetic variation. The US Food and Drug Administration (FDA) “Table of Pharmacogenomic Biomarkers in Drug Labeling” contains 457 entries (status March 2021) relating to dosage and administration, warnings, precautions, drug interactions, adverse reactions, or clinical pharmacology [1]. For example, codeine, a frequently prescribed opiate present in Tylenol #3 (acetaminophen with codeine), contains the boxed warning:

Death Related to Ultra-Rapid Metabolism of Codeine to Morphine. Life-threatening respiratory depression and death have occurred in children who received codeine. Codeine is subject to variability in metabolism based upon CYP2D6 genotype (described below), which can lead to an increased exposure to the active metabolite morphine. (...) For example, many reported cases of death occurred in the post-operative period following tonsillectomy and/or adenoidectomy, and many of the children had evidence of being ultra-rapid metabolizers of codeine. (...) Nursing Mothers: At least one death was reported in a nursing infant who was exposed to high levels of morphine in breast milk because the mother was an ultra-rapid metabolizer of codeine. Breastfeeding is not recommended during treatment with Codeine Sulfate Tablets.

A survey involving clinicians from academic medical centers showed 99% agreed that PGx variants would influence a patients’ response to drug therapy and should be acted upon when a clinically significant drug-genome interaction was present (92%) [2]. Previous studies have shown that over 80% of patients can carry at least one functional gene variant influencing one of the 100 most prescribed medications in the United States, and the rate of rehospitalization can be significantly reduced by implementation of PGx test recommendations [3-7].

Recommendations for actionable prescribing decisions are routinely based on clearly defined, peer-reviewed guidelines with different evidence levels (levels 1-4) issued by international pharmacogenetic consortia and professional societies such as the Clinical Pharmacogenetics Implementation Consortium (CPIC) and maintained in high-quality public and expert-curated databases, including PharmGKB [8-11]. Currently, most laboratories conducting PGx testing use targeted genotyping technologies to screen for specific variants to determine ADRs. Examples of these technologies include single or multiplexed polymerase chain reaction (PCR) assays combined with Taqman hydrolysis probe chemistry, microarrays (ThermoFisher Scientific), mass spectrometry (Agena Biosciences), bead-based molecular assays (Luminex), or next-generation sequencing (NGS) assays (Illumina) [12-14]. In 2018, Fabbri et al [15] described 38 commercially available PGx test panels offering personalized medication prescription guidance in clinical settings. The only genes included in all of these panels were *CYP2D6* and *CYP2C19*. Of the 38 panels, 31 (82%) included 8 genes or less [15]. PGx testing as described in this study encompasses deep sequencing (>1000X) of 141 single-nucleotide polymorphisms (SNPs) or indels across 23 genes by NGS.

**Figure 1:**
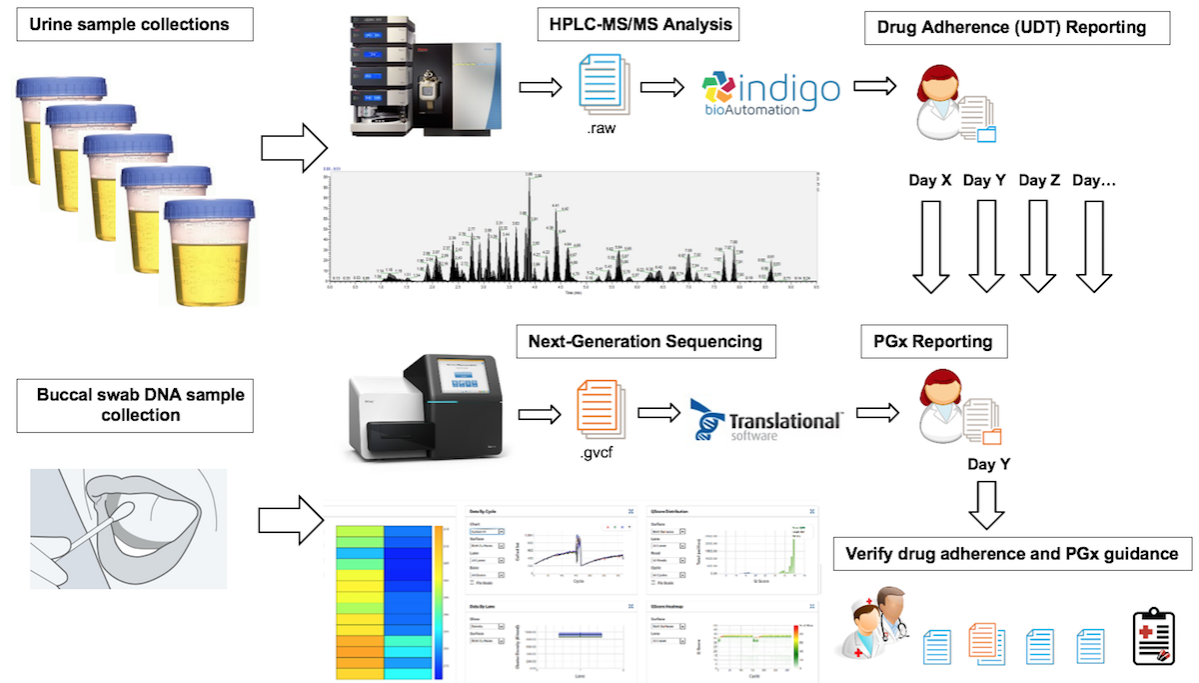
Overview of this study to determine the implementation of PGx report recommendations as compared to urine drug adherence reports in a pain management setting after application of a deep sequencing PGx panel. PGx: pharmacogenetic; UDT: urine drug toxicology.

The aim of this study is to evaluate the overall use and to describe how PGx report recommendations, including genetic-based dosing guidance (PGx), drug-gene interaction (DGI)–based guidance, and drug-drug interaction (DDI)–based guidance, were applied to optimize drug dosing in a clinical setting that had not previously relied on pharmacogenetic test reports. Changes in prescription, patient compliance, and drug use were monitored based on updated medication lists and data in associated quantitative urine drug toxicology (UDT) reports, with limited access to patient progress reports. UDT reports were evaluated in a pain management setting before and after application of PGx panels to prevent ADR events (Figure 1).

## Methods

### Overview

This study was conducted in accordance with the Declaration of Helsinki with written informed consent from each patient. Patient data collection and summaries at Alcala Testing and Analysis Services (ATAS) were approved by the Alcala Pharmaceutical Inc Institutional Review Board (IORG0010127, IRB00012026, #R003). All test samples derived from human patients were deidentified of their health information as defined by Health Insurance Portability and Accountability Act guidelines. Patient data for comparison of urine drug adherence testing before and after PGx reporting, with limited access to patient progress notes, were obtained retrospectively from patients (n=171) in a pain management clinic representing a patient population from 2016 to 2018 within the western United States. While no patient demographics data were available, the Results section shows the genotype frequencies of the “San Diego cohort” (SDC) of this study compared to 5 super populations from the 1000 Genomes Database: African (AFR), South Asian (SAS), Ad Mixed American (AMR), East Asian (EAS), and European (EUR). Pearson correlation analysis (Multimedia Appendix 1) showed the “SDC” positively correlates to all allele frequencies in the 1000 Genomes Database (ALL=0.76; *P*=1.019 × 10^–11^). SDC (n=171) closely correlates to the AMR (0.77), EUR (0.78), and SAS (0.78) super populations but is less representative of the EAS (0.54) and AFR (0.55) population frequencies. Other available data included deidentified pre- and post-PGx medication lists, PGx, and urine drug adherence data (see sections PGx Dosing/DGI/DDI Data Interpretation and Reporting, and Drug Adherence Testing).

### Genes

A total of 23 genes were included in the described PGx panel at the time of design in April 2016 (*ADRA2A*, *CES1*, *COMT*, *CYP1A2*, *CYP2C19*, *CYP2C9*, *CYP2D6*, *CYP3A4*, *CYP3A5*, *DRD1*, *DRD2*, *F2*, *F5*, *GNB3*, *HTR1A*, *HTR2A*, *HTR2C*, *MTHFR*, *OPRM1*, *SLC6A2*, *SCL6A4*, *SLCO1B1*, and *VKORC1*) to include the most up-to-date guidance covering 198 drugs with a major emphasis on pain, psychiatry, and addiction medicine as described in the section PGx Dosing/DGI/DDI Data Interpretation and Reporting.

### Selection of Target Regions

The online probe design was performed by entering target regions into Design Studio software (Illumina) [16]. Unique reference SNP cluster ID (rsID) numbers were assigned per target coordinate and region. A total of 79 target regions (defined across start and stop coordinates, see Multimedia Appendix 2) covering 141 SNPs or indels were covered by 82 amplicons with an average amplicon size of 250 base pairs (bp) across 23 genes. Multiple target regions covering multiple rsIDs were targeted across each gene (eg, 27 rsIDs within *CYP2D6*; see Multimedia Appendix 2). Possible gaps in target coverages, repeats, and GC-rich regions that could interfere with optimal amplification of all desired regions were identified in 3 iterations (design 32844, 32865, and 98659) and optimized for TruSeq Custom Amplicon Low Input (TSCA-LI) assay technology (*Homo sapiens* [UCSC hg19]; variant source: 1000 Genomes). Predicted coverage of the full region of interest was 100% with all amplicons showing scores at 100%. Oligonucleotide probes were synthesized and pooled at Illumina (San Diego, CA) into a Custom Amplicon Tube.

### DNA Isolation and Genotyping

Genomic DNA (gDNA) was isolated from up to 4 buccal swab specimens provided by the pain management clinic using PureLink Genomic DNA Isolation (ThermoFisher Scientific, Carlsbad, CA) and Agencourt DNAdvance Genomic DNA Isolation kits (Beckman Coulter, Indianapolis, IN). Quality and concentration of gDNA were determined using Qubit 3.0 Fluorometric Quantitation (ThermoFisher Scientific). NGS was carried out on a MiSeq system (Illumina, San Diego, CA) with 2 × 150 bp paired-end reads using the TruSeq Custom Amplicon v1.5 Targeted Resequencing workflow (Illumina) for up to 24 samples per plate. HYB and EXT_LIG programs were as described in the TSCA-LI protocol. Amplification was carried out at 32 cycles (<96 amplicon plexity). After cleanup and normalization by AMPure XP magnetic beads, pooled libraries were denatured at 98 °C for 2 minutes and cooled on ice for 5 minutes. Denatured PhiX control (12.5 pmol/L) was spiked into the library pool at 1% and loaded onto an Illumina MiSeq instrument at 7 pmol/L for automated cluster generation and sequencing according to the manufacturer’s instructions. All targets and 50 bp flanking regions were sequenced, the capture region totaled approximately 20 kb.

### Data Analysis

The TruSeq Amplicon workflow version 1.0.0.61 on the MiSeq instrument was used to perform primary analysis by Real Time Analysis (RTA; version 1.18.54) during the sequencing run. Base calls of indexed raw sequence reads and demultiplexing were performed using bcl2fastq. MiSeq Reporter version 2.6.2.3 performed secondary analysis on base calls and quality scores generated on-instrument by the RTA software and evaluated short regions of amplified DNA for variants. Clusters from each sample were aligned against amplicon sequences from the provided manifest file (Design 98659). The first read was evaluated against the probe sequence for each amplicon in the manifest, which is the reverse complement of the downstream locus-specific oligo (DLSO). If the start of the read matches (with at most 1 mismatch) a probe sequence, the read was aligned against the target or targets for that probe sequence. If no such match was found for the read, MiSeq Reporter checked for any probe sequence that was matched with fewer than six mismatches and attempted to align against these amplicons. For paired-end data, the second read was handled similarly, except that read 2 was compared to upstream locus-specific oligo (ULSO) sequences. After the probe sequence (ULSO or DLSO) was matched, adapter sequences were removed, and trimmed reads were mapped to the human reference genome (GRCh37 hg19) using banded Smith-Waterman alignment generated in the .bam file format. The maximum indel length is normally 10 bp but was overridden using the sample sheet setting CustomAmpliconAlignerMaxIndelSize set to 250 (higher values improve indel sensitivity but impact workflow speed). Other sample sheet settings included IndelRepeatFilterCutoff set to 1, MinimumCoverageDepth=1, VariantMinimumGQCutoff=1, VariantFilterQualityCutoff=1, VariantCaller=GATK, VariantAnnotation=MARS, and outputgenomevcf=TRUE. Genome Analysis Toolkit (GATK, Broad Institute) identifies variants and writes .vcf and .gvcf output files to the Alignment folder. SNPs and short indels were identified using GATK for each sample, and false discovery rates for each variant were evaluated using coverage (read depth), the Qscore (quality), and the GQX value (a conservative measure of genotype quality derived from the minimum of the GQ and QUAL values listed in the .vcf file). The Qscore predicts probability of an erroneous base call (Q20 represents the probability to call an erroneous base out of 100, reflecting an accuracy of the sequenced base at 99%, Q30=99.9%, Q40=99.99%, etc). Coverage for a defined region is the total number of reads passing quality filters at this position representing a given nucleotide. Only variants showing Qscores and GQX values >30 and coverage >100X were considered in this study. The average coverage per target exceeded 2000X. Two positive gDNA controls (PC1 and Coriell cell line NA19920 gDNA) and one negative (RS1 buffer) control were sequenced per plate (up to 48 samples). All 167 mutation sites covering 141 SNPs, 2 sex probes, and 1 indel (43-44 bp insertion in the *SLC6A4* promoter region—short [S] or long [L] form—see Multimedia Appendix 2) within the 23 genes identified by MiSeq Reporter were reviewed for each sample in VariantStudio software (Illumina) assisted by the PASS filter function. Gender (SRY) probes were matched to the provided gender in the sample requisition.

### Copy Number Variation and Indel Assays

Copy number variations (CNVs) of *CYP2D6* were identified with two different PCRs for detection of CYP2D6*XN duplication or CYP2D6*5 deletion events by long-range PCR as previously described [17,18]. A total of 10 nanograms of input gDNA was used with Takara LA *Taq* polymerase (Takara Bio USA, San Diego, CA) carried out according to the manufacturer’s instructions. The long-range PCR conditions for duplication testing were as follows: initiation at 94 °C for 2 minutes, 27 cycles of 98 °C for 20 seconds, 61.4 °C for 20 seconds and 68 °C for 10 minutes, and termination at 72 °C for 10 minutes. PCR conditions for deletion tests were the same except annealing was at 65 °C for 25 seconds and extension at 68 °C for 5 minutes with 25 cycles and termination at 72 °C for 6 minutes. Long-range PCR products were analyzed by 1% agarose gel electrophoresis. The presence of a 10 kB fragment (by primers CY_DUP_5 and CY_DUP_3) indicated duplicated or multicopy *CYP2D6* alleles and a 3.5 kb product (by primers CY_DEL_5 and CY_DEL_3) was indicative of the deletion (CYP2D6*5 allele). Amplification of the S and L variant of the 5-HTT gene-linked polymorphic region (5-HTTLPR) of *SLC6A4* was accomplished with oligonucleotide 5-HTTF, corresponding to nucleotide positions −1346 to −1324 and 5-HTTR (positions from −910 to −888) as previously described [19,20], except amplification was performed in 25 μl containing 10 ng of gDNA, 1.5 mM MgCl_2_, 200 μM dNTPs, 1X Colorless GoTaq Flexi buffer, 0.4 μM of each primer, and 1 U of Hot Start GoTaq DNA polymerase (Promega Biosciences, San Luis Obispo, CA). Initial denaturation was performed at 98 °C for 3 minutes, followed by 35 cycles at 94 °C for 1 minute, 64 °C for 30 seconds, and 72 °C for 2 minutes. PCR products were resolved by 2% agarose gel electrophoresis. A total of 458 and 415 bp fragments indicated the L/S genotype for *SLC6A4*; single 415 bp bands or 458 bp bands (no double band profile) indicated the S/S and L/L genotypes, respectively. All primer sequences are listed in Multimedia Appendix 3.

### PGx Dosing/DGI/DDI Data Interpretation and Reporting

**Figure 2:**
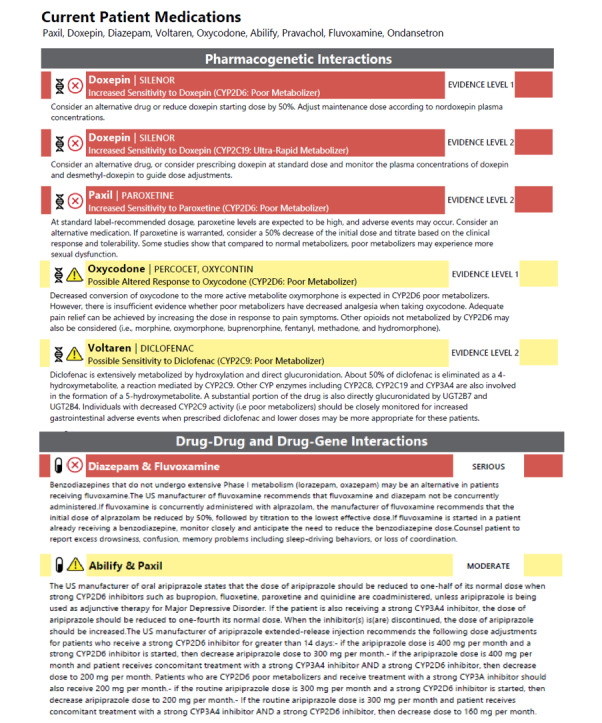
Example of pharmacogenetic (PGx) report results showing PGx dosing guidance (ie, based solely on genetic metabolizer status categories: “Normal Metabolizer,” “Poor Metabolizer,” “Intermediate Metabolizer,” and “Ultra-rapid Metabolizer”), as well as drug-gene interactions (DGIs) and drug-drug interactions (DDIs). Evidence level 1 descriptions were actionable with established evidence-based clinical guidelines issued by international PGx consortia, professional societies, or regulatory bodies (Clinical Pharmacogenetics Implementation Consortium, Dutch Pharmacogenetics Working Group, Food and Drug Administration, European Medicines Agency, Canadian Pharmacogenomics Network for Drug Safety, American College of Medical Genetics and Genomics). Evidence level 2 descriptions were informative, requiring further investigations. PGx dosing guidance, DGIs, and DDIs were further marked as either yellow (moderate) or red (serious) interactions (also see Multimedia Appendix 4).

All samples and positive controls were imported as .gvcf files into a customized portal through Translational Software Inc (TSI, Bellevue, WA) [21]. Specifically, to accommodate reporting based on 23 genes, 141 SNPs or indels, and associated haplotypes newly combined in this panel (Multimedia Appendix 2), TSI bioinformaticians collaborated with ATAS scientists to include the most up-to-date guidance across 2 evidence levels for PGx dosing and DDIs (Figure 2). Recommendations from six different international pharmacogenetic consortia, professional societies, or regulatory bodies (CPIC, Dutch Pharmacogenetics Working Group, FDA, European Medicines Agency, Canadian Pharmacogenomics Network for Drug Safety, and American College of Medical Genetics and Genomics) were incorporated in the reporting algorithm. Integrated recommendations covered 13 drug categories and 198 drugs with a major emphasis on pain, psychiatry, and addiction medicine drugs (Multimedia Appendix 4).

After portal entry of *SLC6A4* indel S/S, La/La, La/Lg, or Lg/Lg variants and *CYP2D6* deletion or duplication, data transfer of all variants and phenotype calls were reviewed for samples and quality controls prior to medical report generation for each patient. Translational Software provides interpretations of specific variants for “PGx DOSING” guidance (ie, based solely on genetic metabolizer status categories: “Normal Metabolizer,” “Poor Metabolizer,” “Intermediate Metabolizer,” or “Ultra-rapid Metabolizer”) and DGI or DDI warnings provided by a third-party agreement with First Databank (FDB). Control gDNA from NA18861, NA18868, NA19920, and NA19226 purchased from Coriell Cell Biorepositories and internal positive controls were used for validation of the TSCA-LI workflow with design 98659, CNV/indel assay validations, and for the evaluation of the data interpretation software by TSI.

### Drug Adherence Testing

All PGx reports were compared to urine toxicology reports generated before or after clinicians received the PGX report. Urine toxicology reports reviewed by clinical laboratory scientists with ASCENT review software (IndigoBio Automation) [22] were made available by routine HPLC-MS/MS presumptive and confirmatory urine drug testing at ATAS from 2016 to 2018 [23].

## Results

Analytical sensitivity (call rate) was determined at >97.1% by positive agreement of all 141 variants including sex determination through 2 SRY probes and CNVs/indels. Genomic DNA ranging from 0.64 to 26 ng/µL (5-195 ng input gDNA) was sequenced across three validation plate runs with 68 positive control samples showing unambiguous genotypes. Buccal swabs were stored for up to 14 days at 4 °C prior to gDNA preparation; gDNA storage stability at 4 °C was confirmed for up to 6 days and up to 6.5 months for storage at –20 °C with up to 10 freeze/thaw cycles to yield high quality (>99.3%) genotyping results Multimedia Appendix 5.

**Figure 3:**
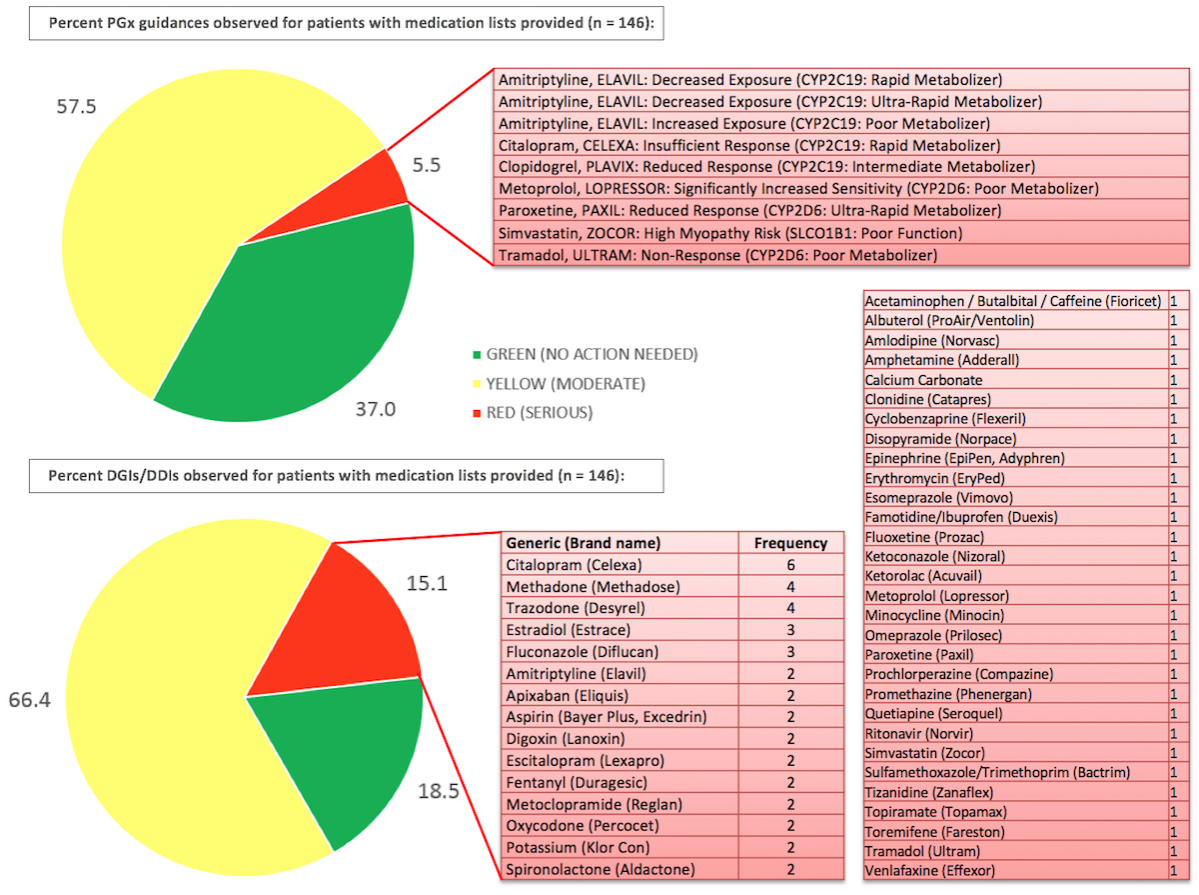
Percent PGx dosing guidance, DGIs, and DDIs observed for patients with medication lists provided (n=146) sorted by the expected normal response to a drug based on PGx metabolizer status or no interaction observed for DGIs/DDIs. Green: no action required; yellow (moderate) or red (serious) interactions prompt actionable PGx or DGI/DDI recommendations. Specific drug names and the associated genotype for PGx dosing or frequency for DGIs/DDIs are shown for serious cases. DDI: drug-drug interaction; DGI: drug-gene interaction; PGX: pharmacogenetic.

All alleles covered per gene target or targets and resulting phenotypes were routinely described in the test details section in each PGx report (Table 1) following the results for PGx dosing and DGI or DDI (Figure 2). Of the 171 patients studied, drug adherence data was not available for 69 patients for which PGx report data was summarized. PGx report implementation could only be studied on the remaining 102 patients. A total of 26 PGx reports showed no medication list provided by the clinic, 8 of which medication lists were made available and added onto the PGx report retroactively. Medication lists provided showed that patients were prescribed an average of 5 different medications (ranging from 0 to 25 medications), resulting on average in 1 moderate pharmacogenetic guidance and 3 moderate DDI observations per patient. Among patient PGx reports with medication lists provided (n=146), 57.5% (n=84) showed one or more moderate and 5.5% (n=8) at least one serious PGx (ie, purely gene-based) interaction. A total of 96 (66%) patients showed at least one moderate and 15% (n=22) one or more serious DGIs or DDIs (Figure 3 and Multimedia Appendix 6).

**Table 1:** Example of pharmacogenetic report test detail summaries and alleles covered.

Gene	Genotype	Phenotype	Alleles tested
*CYP2C9*	*1/*1	Normal metabolizer	*2, *3, *4, *5, *6, *7, *8, *11, *14, *27
*CYP2C19*	*2/*17	Intermediate metabolizer	*2, *25, *3, *4, *4B, *5, *6, *7, *8, *9, *10, *12, *14, *15, *17
*CYP2D6*	*1/*2	Normal metabolizer	*2, *3, *31, *33, *4, *4M, *46, *49, *53, *6, *7, *8, *9, *10, *11, *12, *14A, *14B, *15, *17, *29, *35, *38, *41, *44, *5 (gene deletion), XN (gene duplication)
*CYP3A5*	*3/*3	Poor metabolizer	*1D, *2, *3, *3B, *3C, *4, *6, *7, *8, *9
*CYP3A4*	*1/*1	Normal metabolizer	*2, *4, *5, *8, *11, *12, *13, *16A, *16B, *17, *18A, *18B, *20, *22
*VKORC1*	-1639G>A A/A	High warfarin sensitivity	-1639G>A, 1542G>C, 5808T>G, 1173C>T, rs11540137, rs13337470, 698C>T, 2255C>T, 3730G>A
*CYP1A2*	*1F/*1F	Normal metabolizer—higher inducibility	*1C, *1D, *1E, *1F, *1J, *1K, *1L, *1V, *1W, *7
*SLCO1B1*	521T>C T/C	Decreased function	388A>G, 521T>C, 467A>G, -11187G>A, 1865+248G>A
*COMT*	Val158Met A/G	Intermediate *COMT* activity	Val158Met
*OPRM1*	A118G A/A	Normal *OPRM1* function	A118G
*HTR2C*	-759C>T C/T	Heterozygous for the C allele (rs3813929)	-759C>T, 2565G>C
*SLC6A4*	S/La	Decreased serotonin transporter expression	La, S, Lg
*ADRA2A*	C-1291G C/C	Homozygous for C allele	C-1291G
*SLC6A4*	463T>G A/A	Homozygous for A allele	La, S, Lg
*HTR2A*	rs7997012 G/G	Homozygous for G allele (rs7997012)	102C>T, -1483G>A, rs7997012
*HTR2C*	2565G>C C/C	Homozygous for C allele (rs1414334)	-759C>T, 2565G>C
*HTR2A*	-1438G>A, T/T	Homozygous for T allele (rs6311)	102C>T, -1483G>A, rs7997012
*DRD2*	-241A>G, T/C	Heterozygous for rs1799978 C allele	-241A>G, rs2283265, 939T>C, 957C>T
*DRD2*	rs2283265 C/C	Homozygous for rs2283265 C allele	-241A>G, rs2283265, 939T>C, 957C>T
*MTHFR*	1298A>C AA 677C>T CC	No increased risk of hyperhomocysteinemia	677C>T, 1298A>C, 1305C>T
*MTHFR*	677C>T CC	Normal *MTHFR* activity	677C>T, 1298A>C, 1305C>T
Factor II Factor V Leiden	20210G>A GG 1691G>A GG	No increased risk of thrombosis	20210G>A, 1691G>A

Phenotypes and associated genotypes are summarized in Table 2 with an overview of population frequencies compared to the SDC. As shown in Figure 3, 5.5% (n=8) of 146 patients showed serious ADRs based on changes in either *CYP2C19* (poor, intermediate to rapid metabolizers) or *CYP2D6* (poor or ultra-rapid metabolizers) and one *SLCO1B1* reduced function genotype. *CYP2C19* genotype frequencies for 3 metabolizer types causing serious ADRs were spread across all 5 super populations ranging from 0.9% to 47.4% frequency (Table 2, *CYP2C19* section). *CYP2D6* genotype frequencies for intermediate to ultra-rapid metabolizers ranged from 1.2% to 57.1% frequency and *SLCO1B1* poor function genotypes from 1.8% to 37% (Table 2, *CYP2D6* and *SLCO1B1* section). While SAS population frequencies for *CYP2C19* ultra-rapid metabolizers and *CYP2D6* poor metabolizers are determined as nonexistent in the 1000 Genome Database data, more recent studies show frequencies of 0.24% [24] and 0.84% [25], indicating possible occurrence within the SAS super population.

**Table 2:** Observed phenotypes and associated genotypes with an overview of population frequencies compared to this study (N=171).

Gene		Phenotype/functional status	Defining variant	Genotype(s)	Genotype frequencies^a^						
					Super population frequency (1000 Genomes Project; %)						This study (%)
					All	AFR^b^	AMR^c^	EUR^d^	EAS^e^	SAS^f^	SDC^g^
**Adrenoceptor alpha 2A**											
	*ADRA2A*	Homozygous for G allele	Ancestral: G	G/G	33.3	51.9	13.3	6.4	47.6	35.6	15.9
	*ADRA2A*	Heterozygous for the G allele	rs1800544 (C-1291G)	G/C	42	39	45	39.6	44	44.4	30.7
	*ADRA2A*	Homozygous for C allele	rs1800544 (C-1291G)	C/C	24.6	9.1	41.8	54.1	8.3	20	53.4
**Catechol-O-methyltransferase**											
	*COMT*	High/normal COMT activity	Ancestral: G	G/G	41.3	52.6	36.9	26.4	52.4	32.9	21.6
	*COMT*	Intermediate COMT activity	rs4680 (1947 G>A, Val158Met)	G/A	43.6	38.6	50.7	47.1	39.3	46	65.1
	*COMT*	Low COMT activity	rs4680 (1947 G>A, Val158Met)	A/A	15.1	8.8	12.4	26.4	8.3	21.1	13.4
**Cytochrome P450 family 1 subfamily A member 2**											
	*CYP1A2*	Normal metabolizer: possible inducibility	Ancestral: C or G	*1A/*1A (C/C or G/G), *1A/*1V, *1A/*1W	15.2	19.8	7.5	10.9	11.5	22.7	23.7
	*CYP1A2*	Normal metabolizer: higher inducibility	rs762551 (-163)C>A	*1A/*1F (C/A), *1F/*1F (A/A)	84.8	80.2	92.5	89.1	88.5	77.3	74.2
	*CYP1A2*	Poor metabolizer: lower inducibility	rs2069514 (-3860G > A)	*1A/*1C (G/A)	28.9	44.5	38.9	4	40.3	14.7	1.0
	*CYP1A2*	Unknown phenotype	Multiple^h^	*1L/*1L, *1L/*1W	N/A^i^	N/A	N/A	N/A	N/A	N/A	1.0
**Cytochrome P450 family 2 subfamily C member 19**											
	*CYP2C19*	Normal metabolizer	Ancestral: G or C	*1/*1 (G/G or C/C)	33.7	27.5	57.6	31.8	42.1	18.8	29.5
	*CYP2C19*	Intermediate metabolizer	rs4244285 (19154G>A)	*1/*2 (G/A)	32.8	27.1	19.3	26.6	47.4	41.3	34.4
	*CYP2C19*	Poor metabolizer	rs4244285 (19154G>A)	*2/*2 (A/A)	5.8	3.5	0.9	1.2	7.5	15.1	15.6
	*CYP2C19*	Rapid metabolizer	rs12248560 (-806C>T)	*1/*17 (C/T)	24.7	36.8	20.5	36	3	22.3	20.5
	*CYP2C19*	Ultra-rapid metabolizer	rs12248560 (-806C>T)	*17/*17 (T/T)	3	5.1	1.7	4.4	0	2.5	1.2
**Cytochrome P450 family 2 subfamily C member 9**											
	*CYP2C9*	Normal metabolizer	Ancestral: G or A	*1/*1	90.5	99.5	92.5	85.7	93.5	78.7	77.0
	*CYP2C9*	Intermediate metabolizer	rs1057910 (A/C)	*1/*3 (A/C)	9.3	0.5	7.5	14.1	6.3	20.7	19.7
	*CYP2C9*	Poor metabolizer	rs1057910 (C/C)	*3/*3 (C/C)	0.2	0	0	0.2	0.2	0.6	3.3
**Cytochrome P450 family 2 subfamily D member 6**											
	*CYP2D6*	Normal metabolizer	Ancestral: multiple	*1/*1, *1/*2, *1/*4, *1/*5	71.5	82.8	82.3	88.0	41.5	79.6	89.7
	*CYP2D6*	Intermediate metabolizer	*10 - rs1065852 (100C>T)	*5/*10, *10/*15, *4/*17, *4/*29, *4/*41	23.8	11.3	14.8	5	57.1	16.5	4.1
	*CYP2D6*	Poor metabolizer	*4 - rs3892097 (1846G>A)	*4/*4, *4/*5 (A/A)	2.1	1.2	2.9	4.6	0	2.5	3.7
	*CYP2D6*	Ultra-rapid metabolizer^j^	XN (Duplication, XN Exon 9)	*1/*2 XN, *1/*4 XN, *1/*35 XN	2.64	4.66	N/A	2.37	1.37	1.37	2.5
**Cytochrome P450 family 3 subfamily A member 4**											
	*CYP3A4*	Normal metabolizer	Ancestral: G	*1/*1 (G/G)	97	99.8	94.8	90.3	100	98.8	67.6
	*CYP3A4*	Intermediate metabolizer	rs35599367 (intron 6 C>T)	*1/*22 (G/A)	3	0.2	5.2	9.7	0	1.2	28.4
**Cytochrome P450 family 3 subfamily A member 5**											
	*CYP3A5*	Normal metabolizer	Ancestral: T	*1/*1 (T/T)	22.7	67.6	5.8	0.4	7.9	12.1	5.3
	*CYP3A5*	Intermediate metabolizer	rs776746 (6986A>G)	*1/*3 (T/C)	30.3	28.7	29.1	10.5	41.5	42.3	45.8
	*CYP3A5*	Poor metabolizer	rs776746 (6986A>G)	*3/*3 (C/C)	47	3.6	65.1	89.1	50.6	45.6	54.2
**Dopamine receptor D2**											
	*DRD2*	Homozygous for rs1799978 C allele	Ancestral: C	C/C	2.2	4.1	0.3	0.6	1	0.8	1.3
	*DRD2*	Heterozygous for rs1799978 C allele	rs1799978 (-241A>G)	T/C	19.5	26.5	14.7	10.7	28	13.7	12.3
	*DRD2*	Homozygous for rs1799978 T allele	rs1799978 (-241A>G)	T/T	78.4	69.4	85	88.7	68.3	85.5	86.4
	*DRD2*	Homozygous for rs2283265 C allele	Ancestral: C	C/C	62	85	55.9	73.6	34.3	51.9	41.6
	*DRD2*	Heterozygous for rs2283265 A allele	rs2283265 (724-353G>T)	C/A	30.6	13.8	35.2	23.3	48.4	39.1	32.4
	*DRD2*	Homozygous for rs2283265 A allele	rs2283265 (724-353G>T)	A/A	7.4	1.2	8.9	3.2	17.3	9	26.1
**5-hydroxytryptamine receptor 2A (Serotonin 2A receptor gene)**											
	*HTR2A*	Homozygous for G allele (rs7997012)	Ancestral: G	G/G	56.2	97.1	41.8	33.2	56.7	34.2	7.2
	*HTR2A*	Heterozygous for the A allele (rs7997012)	rs7997012 (614-2211T>C)	A/G	33.1	2.9	46.1	47.7	34.9	47.6	30.8
	*HTR2A*	Homozygous for the A allele (rs7997012)	rs7997012 (614-2211T>C)	A/A	10.7	0	12.1	19.1	8.3	18.2	62.0
	*HTR2A*	Homozygous for the C allele (rs6311)	Ancestral: C	C/C	32.4	36	40.1	33	18.3	36	25.2
	*HTR2A*	Heterozygous for the T Allele (rs6311)	rs6311 (-1438G>A)	C/T	46.5	46.1	47.6	46.5	45.8	47	63.8
	*HTR2A*	Homozygous for the T allele (rs6311)	rs6311 (-1438G>A)	T/T	21.1	17.9	12.4	20.5	35.9	17	11.0
**5-Hydroxytryptamine receptor 2C (serotonin 2C receptor gene)**											
	*HTR2C*	Homozygous for the C allele (rs1414334)	Ancestral: C	C/C	35	15.7	41.2	39	50.2	40.3	52.5
	*HTR2C*	Heterozygous for the C allele (rs1414334)	rs1414334 (2565G>C or 114138144C>G)	G/C	51.9	49.4	53.4	51.3	49.2	54.4	41.9
	*HTR2C*	Homozygous for the G allele (rs1414334)	rs1414334 (2565G>C or 114138144C>G)	G/G	13.1	34.9	5.4	9.7	0.6	5.3	5.5
	*HTR2C*	Homozygous for the C allele (rs3813929)	Ancestral: C	C/C	88.1	98.2	82.1	85.5	85.5	78.1	85.5
	*HTR2C*	Heterozygous for the C allele (rs3813929)	rs3813929 (-759C>T)	T/C	10.7	1.8	17.9	13.5	13.5	17.6	12.8
	*HTR2C*	Homozygous for the T allele (rs3813929)	rs3813929 (-759C>T)	T/T	1.2	0	0	1	1	4.3	1.7
**Opioid receptor mu 1**											
	*OPRM1*	Normal OPRM1 function	Ancestral: A	A/A	62.5	98.2	64.6	70.2	36.7	31.3	53.0
	*OPRM1*	Altered OPRM1 function	rs1799971 (A118G)	A/G	30.4	1.8	30.8	27.2	48	53.8	47.0
	*OPRM1*	Altered OPRM1 function	rs1799971 (A118G)	G/G	7.1	0	4.6	2.6	15.3	14.9	0.0
**Solute carrier family 6 member 4**											
	*SLC6A4*	Homozygous for C allele	Ancestral: C	C/C	28.7	3.8	30.5	17.9	68.4	31	16.3
	*SLC6A4*	Heterozygous for the C allele	rs1042173 (463T>G C/A)	C/A	39.7	29.5	47.3	51.7	27.6	48.3	64.8
	*SLC6A4*	Homozygous for A allele	rs1042173 (463T>G C/A)	A/A	31.6	66.7	22.2	30.4	4	20.7	18.9
	*SLC6A4*	Normal serotonin transporter expression	5-HTTLPR (L/S) and rs25531 (A/G)	La/La (L'L' group^k^)	N/A	27	22	25	8	8	24.7
	*SLC6A4*	Decreased serotonin transporter expression	5-HTTLPR (L/S) and rs25531 (A/G)	La/Lg, La/S (L'S' group^k^)	N/A	49	51	50	30	30	43.8
	*SLC6A4*	Low serotonin transporter expression	5-HTTLPR (L/S) and rs25531 (A/G)	Lg/Lg, Lg/S, S/S (S'S' group^k^)	N/A	24	27	25	62	62	31.5
**Solute carrier organic anion transporter family member 1B1**											
	*SLCO1B1*	Normal function	Ancestral: T	T/T	43.5	72.9	27.4	15.1	57.1	30.3	81.6
	*SLCO1B1*	Decreased function	rs4149057 (521T>C)	T/C	39.5	25.3	44.4	48.3	37.1	48.9	16.8
	*SLCO1B1*	Poor function	rs4149057 (521T>C)	C/C	17	1.8	28.2	36.6	5.8	20.8	1.6
**Vitamin K epoxide reductase complex subunit 1**											
	*VKORC1*	Low warfarin sensitivity	Ancestral: G	G/G	50.9	89.5	35.2	38.2	1.8	73.4	49.8
	*VKORC1*	Intermediate warfarin sensitivity	rs9923231 (-1639G>A)	G/A	27.1	10	47.6	46.1	19.4	24.1	39.7
	*VKORC1*	High warfarin sensitivity	rs9923231 (-1639G>A)	A/A	22	5	17.2	15.7	78.8	2.5	10.5

^a^The frequencies for this table were referenced from the 1000 Genomes Database Ensembl [26]. Further information is available at the Human CYP Allele Nomenclature Database [27]. Populations have been divided into 5 super populations (AFR, SAS, AMR, EAS, and EUR) and this study (SDC).

^b^AFR: African.

^c^AMR: Ad Mixed American.

^d^EUR: European.

^e^EAS: East Asian.

^f^SAS: South Asian.

^g^SDC: San Diego cohort.

^h^See Soyama et al [28].

^i^N/A: not applicable.

^j^Based on Beoris et al [29].

^k^Group definition as per Pascale et al [20]. Population frequencies for *SLC6A4* 5-*HTTLPR* (L/S), rs25331 (A/G) derived from Haberstick et al [30].

Medications affecting patients most severely based on their individual genotype in this cohort were amitriptyline for decreased exposure among 2 *CYP2C19* rapid metabolizers and increased exposure for 1 *CYP2C19* poor metabolizer, citalopram (insufficient response, *CYP2C19* rapid metabolizer), clopidogrel (reduced response, *CYP2C19* intermediate metabolizer), metoprolol with significantly increased sensitivity for a *CYP2D6* poor metabolizer, paroxetine (reduced response in *CYP2D6* ultra-rapid metabolizer), simvastatin (poor function of *SLCO1B1* inducing high myopathy risk), and tramadol (*CYP2D6* poor metabolizer with risk for no response). The top 15 medications affecting patients based on a DGI or DDI were identified (Figure 3). The most frequently occurring moderate DDI involved opioids observed in combination with central nervous system depressants such as muscle relaxants, benzodiazepines, sleep drugs, or the nerve pain medications gabapentin and pregabalin (Multimedia Appendix 6).

Prescription regimens were determined for 102 patients based on drug adherence report data before and after the PGx report was made available. Remaining patients either showed no drug adherence data or limited drug adherence data before the PGx report but no further information afterward. An active change in prescriptions based on the PGx report was observed for 85 (83%) patients for which a specific drug was either discontinued or switched within the defined drug classes of the report, or a new drug added. A total of 17 (17%) patient reports showed no predictive evidence of ADRs even when prescribed up to 11 medications (on average 2.5 medications per patient). Appropriately, no action was taken by the provider in these cases to deviate from the original prescription regimen. All adjustments made to patient prescriptions were studied for potential contraindications or possible new ADRs based on the PGx report.

Of the 85 patients whose medication lists were adjusted, only 3 showed that recommendations in the PGx report were not being followed for unknown reasons. “Patient A” was shown to be administered 5 medications (Keflex, Pennsaid, Skelaxin, MS Contin, and Lidocaine CV). PGx reporting indicated a normal PGx response and one moderate DDI to MS Contin (morphine) and Skelaxin (metaxalone), and a moderate PGx interaction for Pennsaid (diclofenac). Cessation of Skelaxin and Pennsaid removed all moderate ADRs; however, the addition of Percocet (oxycodone and acetaminophen) was not recommended:

Oxycodone - CYP2D6 Poor Metabolizer. Test results indicate a possible increased risk of therapeutic failure. Monitor for decreased response or may select alternative medication.

The decreased response was alleviated with morphine prescriptions, for which there were no contraindications. Progress notes showed patient A:

has tried to use topical patches but experienced a localized reaction to the adhesive on the patch. Oral pain medication of MS Contin and Percocet is helpful. Patient A notes that some days Patient A does not require the max dose of the Percocet.

Coreg (carvedilol) was added to the prescription regimen causing a moderate PGx warning:

CYP2D6 Poor Metabolizer: Test results indicate an increased risk of dizziness during up-titration. Consider standard prescribing and monitoring practices with careful dose titration.

The addition of Silenor (doxepin) was also contraindicated by the PGx report:

CYP2D6 Poor Metabolizer: Test results indicate an increased risk of adverse effects. Consider an alternative medication or a 50% dose reduction with therapeutic drug monitoring.

In this case the prescribed doxepin dosage was minimal (10 mg/day) according to progress notes. For the treatment of major depression or anxiety, adult oral dosages are initially 75 mg per day. The addition of Wellbutrin (bupropion), Soma (carisoprodol), Topamax (topiramate), and Prilosec (omeprazole) showed no contraindication except a moderate DDI between carisoprodol and morphine. The dose reduction for doxepin and the remaining moderate interaction for carvedilol were acceptable, as carvedilol was discontinued and the appropriate monitoring practices were carried out for patient A.

Similarly, for “Patient B,” 7 medications were listed, which showed a switch from codeine to morphine although no warnings against codeine were indicated (patient *CYP2D6* normal metabolizer status). Instead, a switch to morphine warned:

The patient does not carry the COMT Val158Met variant. The patient may require higher doses of morphine for adequate pain control

Additionally, quetiapine and citalopram could cause a serious DDI (“concurrent use with agents known to prolong the QT interval should be avoided”), and the combinations of opioids with gabapentin prompted to “monitor patients for gabapentinoid-related side effects.” Further investigation into progress notes for patient B showed a suspected allergy or ADR to hydrocodone and oxycodone resulting in “nausea,” possibly explaining the emphasis on morphine and the patient avoiding exposure to other opioids such as codeine, hydrocodone, or oxycodone. An increase in morphine 15 mg immediate release formulation tablets (MSir) was initiated from 3 to 4 times daily, eventually 15 mg MSir 3 times per day with an additional 15 mg MS Contin (extended release) 2 times per day. Patient B:

has tried and failed following medications: anti-inflammatory meds, hydrocodone and oxycodone/oxycontin in the past. Patient reports the medication initiated last office visit has provided better relief in pain, notes oral pain medications in form of MSIR and MS Contin are effective and decreases low back pain by no less than a 60% relief in pain, pain level today is 6/10. Upon questioning patient denies adverse reactions such as euphoria/dysphoria

Monthly reviews of the patient’s condition show:

Denies trouble breathing, shortness of breath, asthma, sleep apnea, seizures, blackouts, trouble with memory, headache, fainting spells, numbness, weakness and tremors.

Patient C was maintained on 10 of 11 initial medications with the appropriate removal of Plavix (clopidogrel) after 2 serious PGx warnings:

Reduced Response to Clopidogrel (CYP2C19: Intermediate Metabolizer) Consider alternative therapy

High Myopathy Risk (SLCO1B1: Poor Function). Simvastatin plasma concentrations are expected to be elevated. Consider avoiding simvastatin and prescribe an alternative statin, or consider prescribing simvastatin at a lower starting dose (20 mg/day). Routine creatine kinase (CK) monitoring is also advised. The FDA recommends against the 80 mg daily dose.

An additional serious DDI for Zocor (simvastatin) and Norvasc (amlodipine) warned:

do not exceed a dosage of 20 mg daily of simvastatin in patients receiving concurrent therapy with amlodipine. If concurrent therapy is deemed medically necessary, monitor patients for signs and symptoms of myopathy/rhabdomyolysis, including muscle pain/tenderness/weakness, fever, unusual tiredness, changes in the amount of urine and/or discolored urine.

After PGx reporting, clopidogrel was no longer observed in medication lists for drug adherence reports, but simvastatin was continued with amlodipine, and 9 moderate DDIs remained, cautioning to “limit the dosages and duration of each drug to the minimum possible while achieving the desired clinical effect.” The only alternative statin without adverse interactions recommended was fluvastatin. Progress notes for patient C showed simvastatin was prescribed less than 80 mg per day as recommended by the FDA in the PGx report at 40 mg per day. Patient C “denies muscle cramp, muscle twitches, muscle wasting, muscle weakness, neck pain, joint swelling. Denies fever, fatigue”; however, patient C eventually reported “muscle pain or tenderness” in the latter part of the 2-year treatment window. Monthly urinalysis screens and blood testing showed no discoloration in urine or abnormal glomerular filtration rates, but the reported muscle pain/tenderness and the combination of reduced *SLCO1B1* gene function with concurrent daily 40 mg simvastatin and 5 mg amlodipine possibly indicated a statin-induced myopathy [31].

## Discussion

Serious ADRs can occur based on incidences of poor, intermediate, rapid, and ultra-rapid metabolizer types in all 5 super populations for prescriptions such as amitriptyline, citalopram or clopidogrel, metoprolol, paroxetine, simvastatin, and tramadol. While PGx cannot predict all ADRs (eg, allergies cannot be detected), dosing guidance and the additional DGI and DDI algorithm provided valuable insight to optimize prescription regimens. Limitations within this retrospective study include the lack of detailed patient demographics associated with UDT and PGx reports, and limited access to progress notes and long-term treatment outcomes. Rather than resorting to 1000 Genome Database population frequencies to characterize the SDC, specific demographics and additional case studies as the three previously presented would allow more comprehensive insights as to the combinatorial effect of prescription drugs among polypharmacy pain management patients.

In summary, the effect of PGx reports newly made available to medical staff in this context seems quite significant as observed by the individual PGx dosing/metabolizer status and DGI and DDI recommendations showing a corresponding modification of the medication regimen for each patient. Preventative action was observed for all serious interactions, and only moderate interactions were tolerated where there may not have been other alternatives. This study demonstrates the predictive value of PGx testing combined with a customized informational report to help improve clinical outcomes, which resulted in a successful application for patients in a pain management setting.

## Appendix

Multimedia Appendix 1 Overall positive correlation (*) between 1000 Genome Database super population groups and the San Diego cohort (N=171).

Multimedia Appendix 2 79 Target regions - start and stop coordinates.

Multimedia Appendix 3 Primer sequences.

Multimedia Appendix 4 Summary of PGX DOSING versus drug-gene or drug-drug (DGI/DDI) interactions covered by the ATAS PGx panel.

Multimedia Appendix 5 Stability data.

Multimedia Appendix 6 Summary of PGx (Pharmacogenetic), DGI (Drug-Gene-Interactions) and DDI (Drug-Drug-Interactions) data and implementation.

## Abbreviations

5-HTTLPR: 5-HTT gene-linked polymorphic region
ADR: adverse drug reaction
AFR: African
AMR: Ad Mixed American
ATAS: Alcala Testing and Analysis Services
bp: base pairs
CNV: copy number variation
CPIC: Clinical Pharmacogenetics Implementation Consortium
DDI: drug-drug interaction
DGI: drug-gene interaction
DLSO: downstream locus-specific oligo
EAS: East Asian
EUR: European
FDB: First Databank
FDA: Food and Drug Administration
GATK: Genome Analysis Toolkit
gDNA: genomic DNA
L: long
NGS: next-generation sequencing
PCR: polymerase chain reaction
PGx: pharmacogenomic
rsID: reference single-nucleotide polymorphism cluster ID
RTA: Real Time Analysis
S: short
SAS: South Asian
SDC: San Diego cohort
SNP: single-nucleotide polymorphism
TSCA-LI: TruSeq Custom Amplicon Low Input
TSI: Translational Software Inc
UDT: urine drug toxicology
ULSO: upstream locus-specific oligo
